# Editorial: What Works for Forensic Psychiatric Patients: From Treatment Evaluations to Short and Long-Term Outcomes

**DOI:** 10.3389/fpsyt.2020.615473

**Published:** 2020-11-06

**Authors:** Katarina Howner, Björn Hofvander, James Tapp

**Affiliations:** ^1^Department of Clinical Neuroscience, Centre of Psychiatry Research, Karolinska Institutet, Stockholm, Sweden; ^2^Division for Forensic Psychiatry in Stockholm, Department for Forensic Psychiatry, National Board of Forensic Medicine, Stockholm, Sweden; ^3^Lund Clinical Research on Externalizing and Developmental Psychopathology (LU-CRED), Child and Adolescent Psychiatry, Department of Clinical Sciences Lund, Lund University, Lund, Sweden; ^4^Centre of Ethics, Law and Mental Health (CELAM), Department of Psychiatry and Neurochemistry, Institute of Neuroscience and Physiology, The Sahlgrenska Academy at University of Gothenburg, Gothenburg, Sweden; ^5^Division of Forensic Psychiatry, Region Skåne, Trelleborg, Sweden; ^6^Broadmoor Hospital, West London NHS Trust, Southall, United Kingdom; ^7^Department of Psychology, Kingston University, London, United Kingdom

**Keywords:** forensic psychiatric care, offender, violence, risk assessment, treatment

In many west-world countries the number of psychiatric beds are decreasing while the number of forensic beds are increasing (1, 2). Most forensic psychiatric patients suffer from psychotic disorders, but co-morbidity frequently occurs, such as personality disorders and substance abuse, in combination with violent behavior. For clinicians and staff working with the patients, the role is two-fold, both the psychiatric care for the patient and also preventing re-offending and protecting society. The nature of forensic psychiatric care, and ethical principles that underpin it, make it hard to conduct randomized controlled studies, which can be seen as the gold standard to establishing the efficacy of interventions. In a recent review, evaluating the research within forensic psychiatric care, gaps of knowledge were identified in every aspect of multidisciplinary input (3). This Research Topic encourages researchers to perform and publish high quality research within the field of rehabilitation within forensic psychiatric care.

In a systematic review by Howner et al. the objective was to investigate the effects of pharmacological interventions for forensic psychiatric patients. Only 10 studies met inclusive criteria and most of them were retrospective and non-randomized. Mainly due to the high risk of bias the reliability of the evidence for all outcomes was assessed as very low, and highlights the shortage of knowledge on the effectiveness of pharmacological treatment within forensic psychiatry. Jordan et al. performed a review with the objective to examine if there are biomarkers to support diagnostic process, treatment evaluation, and risk assessment of pedophilic individuals and child sexual offenders. The authors present an overview of the current neurobiological, as well as physiological and psychophysiological approaches to characterize pedophilia and child sexual offending, and then discuss and evaluate the impact of these approaches on the development of biomarkers for diagnosis, therapy and risk assessment in these subjects. The conclusion was that none of the promising parameters is ready to serve as a clinically applicable diagnostic, response or predictive biomarker for pedophilia and child sex offending. The most promising approach seem to be a combination of several measures like EEG, fMRI, eye tracking and behavior. The relative efficacy of The Reasoning and Rehabilitation Program (R&R) and Dialectical Behavioral Therapy–Forensic (DBT-F) was evaluated by Wettermann et al. in a forensic-psychiatric hospital for offenders with substance addiction in Germany. Both programs were associated with improvements in nearly all of the measured constructs, but surprisingly, they did not find superiority for one intervention over Treatment as usual (TAU) or differential effects between the two programs. One-to-One (OTO) is a treatment program based on cognitive-behavioral principles. Berman et al. examined predictive properties of pre- and post- program test scores and background characteristics regarding recidivism, as well as differences between subgroups, in the OTO-program, in Sweden among 776 prisoners shortly awaiting release. The most potent predictor for non-recidivism was program completion, with non-completers 64% more likely to re-offend. Walker and Tulloch performed a qualitative study using semi-structured interviews with 10 nurses working in a high security hospital with forensic psychiatric patients. The purpose of the study was to explore the staffs experience of using mechanical restraints (Soft Restraint Kit), an option in extremely high risk patients allowing other interventions to take place. The conclusion was that Soft Restraint Kits provided a useful risk management method, but prolonged use presents considerable challenges for staff and patient. Preparation, training and supervision were deemed essential. In England, Cornish et al. examined acceptability, feasibility, and practicality in the Forensic Psychiatric and Violence Oxford Tool (FoVOx), a risk assessment tool. In the study, the patient's FoVOx score was compared to clinical risk assessment. In approximately half of the cases the clinical assessment of risk agreed with the FoVOx categories. Clinicians were more likely to provide lower risk categories compared with FoVOx ones. Its use addressed a lack of consistency around risk assessment at the point of discharge and, if used routinely, could assist in clinical decision-making. In Denmark, Bengtson et al. studied rates and facets of long-term violent reoffending in a population of violent offenders who underwent pretrial forensic examinations (FPE). The authors compared the group sentenced to forensic psychiatric care with the group which received ordinary sanctions. The first group was also compared to a group of violent offenders who did not underwent a FPE. During the follow up time FPE examinees; untreated followed by treated, reoffend violently more often than the offenders who did not underwent a FPE. Similar trends are suggested also for severe and recurrent violence suggesting a need for continua-of-services for FPE examinees, independently of medico-legal status (i.e., sentencing to treatment or not). Filicide is tragic and largely understudied, particularly from the perpetrator's perspective. In South Africa, Moodley et al. performed a qualitative study to examine the perceptions of seven women regarding their offenses and their perceptions about their treatment and rehabilitation. Most of the women had been psychotic at the time of the offense, and perceived trauma and regret for their offenses. Support from the community as well as empathy and unconditional positive regard from the staff, notably psychologists and occupational therapists were overwhelmingly present. Forensic psychiatric patients have a reduced life expectancy and Ojansuu et al. aimed to explore to what extent substance abuse disorders accounted for this increased mortality. During the follow-up time a prominent proportion (16%) of all deaths and a majority of the accidental deaths (64%) occurred under the influence of substances. The standardized mortality ratio for the patients with a history of substance abuse disorders was 4.1 compared to 2.8 for those with no such history. The management of substance abuse problems should be one cornerstone of the treatment of patients with both severe mental disorders and substance abuse disorders, and should also be extended to outpatient care. In an opinion article Andiné and Bergman signpost forensic mental health professionals to the importance of interventions that improve brain health, with supporting evidence. They promote that this avenue of interventions should be a future research priority for forensic psychiatric care, given its wide reaching outcome benefits.

## Author Contributions

KH wrote the first draft of the manuscript. BH and JT provided critical revision of the manuscript and important intellectual contributions. All authors read and approved the submitted version.

## Conflict of Interest

The authors declare that the research was conducted in the absence of any commercial or financial relationships that could be construed as a potential conflict of interest.
